# Correction: Length-of-stay and factors associated with early discharge after birth in health facilities in Guinea by mode of birth: Secondary analysis of Demographic and Health Survey 2018

**DOI:** 10.1371/journal.pgph.0005019

**Published:** 2025-08-06

**Authors:** Aline Semaan, Fassou Mathias Grovogui, Thérèse Delvaux, Natasha Housseine, Thomas van den Akker, Alexandre Delamou, Lenka Beňová

In the Introduction, there is an error in the second sentence of the first paragraph. The correct sentence is: This period involves increased risk of maternal and neonatal morbidity and mortality [1].

In the Introduction, there is an error in the second sentence of the fourth paragraph. The correct sentence is: The country carries a high burden of maternal morbidity and mortality, with an estimated maternal mortality ratio of 553 deaths per 100,000 livebirths in 2020 [23]; 34% of which occur in the postnatal period [24].

In the Study Objectives subsection of the Introduction, there is an error in the second sentence of the paragraph. The correct sentence is: The primary objective is to describe postpartum length-of-stay for women who gave birth in a health facility in Guinea in the five-year period (2013–2018), and investigate factors associated with early discharge using Demographic and Health Survey (DHS) data.

In the Study setting subsection of the Methods, there is an error in the fourth sentence of the paragraph. The correct sentence is: Free emergency obstetric care was introduced in all public health facilities in 2010, including for antenatal care (ANC), vaginal birth and caesarean section, which contributed to a significant decrease in unmet obstetric need, as documented in Kissidougou health district [31].

In the Study design, data, population and sample subsection of the Methods, there is an error in the fourth sentence. The correct sentence is: The analysis includes women aged 15–49 years (at the time of the survey) who had their most recent livebirth (i.e., excluding stillbirths) in a health facility in the five years preceding the survey (2013–2018).

In the Outcome and independent variables subsection of the Methods, there is an error in the tenth sentence of the second paragraph. The correct sentence is: We explored additional categories of length-of-stay to describe with nuance the distribution of length-of-stay among those who stayed “too short” (women who had vaginal birth: < 2 hours; 2–3 hours; 4–23 hours; ≥ 24 hours) and those who stayed “too long” (women who had caesarean section: < 24 hours; 24–71 hours; 72–167 hours; ≥ 168 hours).

In the Outcome and independent variables subsection of the Methods, there is an error in the second sentence of the second paragraph. The correct sentence is: We included factors theoretically linked to postpartum length-of-stay and available in the DHS, and spanning five levels: 1-Community & family factors (region, place of residence, ethnicity, marital and cohabiting status, involvement in decision-making regarding own healthcare, number of household members, relationship to head of household); 2-Facility characteristics & norms (type of facility, skilled attendance at birth, birth on weekday/weekend; 3-Women’s socio-economic characteristics (maternal age at birth, education level, occupation at time of survey, household-level wealth index, health insurance and phone ownership, issues perceived as big problems to access healthcare); 4-Women’s needs and obstetric history (parity, antenatal care frequency and timing of first visit, multiple births, whether the pregnancy was wanted, history of previous miscarriage, abortion or stillbirth); and 5-Newborn characteristics (sex, perceived size at birth, newborn survival and time of death).

In the Analysis subsection of the Methods, there is an error in the second sentence of the first paragraph. The correct sentence is: Length-of-stay was described separately by mode of birth (vaginal birth and birth by caesarean section), both as a continuous variable in hours (mean and median), and categorical as early discharge or not (frequencies and percentages).

In the Analysis subsection of the Methods, there is an error in the third sentence of the second paragraph. The correct sentence is: The model for early discharge among women who gave birth by caesarean section was limited to five factors selected based on theoretical grounds because of sample size limitations.

In the Description of the outcome: Pospartum length-of-stay subsection of the Results, there is an error in the first sentence of the third paragraph. The correct sentence is: Fig 2 shows maps with the percentage of women who were discharged early after vaginal birth according to the WHO recommendation of 24 hours (Fig 2A) and the locally-informed cut-off of 6 hours (Fig 2B), in each of the 8 administrative regions in Guinea.

**Fig 2:**
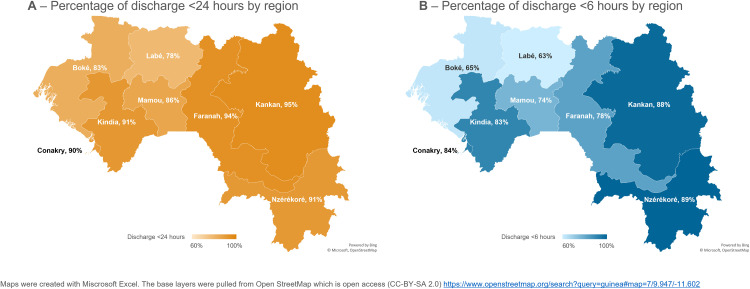
Percentage of women with early discharge according to the WHO recommended (24hrs–Fig 2A) and locally relevant cut-offs (6hrs, Fig 2B), among women who gave their most recent vaginal birth in a health facility in the five-years preceding the Guinea DHS2018 (n = 2,603), by region.

In the Description of the outcome: Pospartum length-of-stay subsection of the Results, there is an error in the fifth sentence of the third paragraph. The correct sentence is: The largest discrepancy between the two cut-offs was in Boke´ with 82.7% [95%CI = 76.1; 87.7] of women discharged early according to WHO recommendations, vs. 64.7% [95%CI = 55.6; 72.9] according to locally-informed cut-off.

In Fig 2, there is an error in the legend of panel B. The number of hours should have been <6 hours instead of <24. Please see the correct Fig 2 here.

In the Discussion, there is an error in the first sentence of the first paragraph. The correct sentence is: This study examined the length-of-stay of postpartum women in health facilities in Guinea.

In the Discussion, there is an error in the sixth and seventh sentences of the first paragraph. The correct sentences are: This study examined the length-of-stay of postpartum women in health facilities in Guinea. This was particularly noted among women who gave vaginal birth, where 30% of those who reported leaving within 2 hours of birth reported not receiving a check. The odds of discharge <6 hours among women who gave vaginal birth were higher for women living in certain regions (e.g., Conakry, Kindia, Kankan and Nzérékoré), for those who gave birth in non-government lower-level facilities and for those who had a singleton birth.

In the Discussion, there is an error in the third sentence of the third paragraph. The correct sentence is: Regional variations in length-of-stay were similarly documented in Cameroon [17], however urban vs rural place of residence did not show a significant association in Guinea; this could be possibly explained by a misclassification bias of areas in the DHS survey between urban and rural, a classification that has beendescribed to be too simplistic and fails to document urbanicity on a spectrum [35].

In the Discussion, there is an error in the third sentence of the ninth paragraph. The correct sentence is: Postpartum women whose newborns were girls had higher odds of staying in the facility the recommended length-of-stay compared to boys, in Guinea similarly to the results of the multi-country analysis [12].

In the Author Contributions, Aline Semaan should be attributed to: Conceptualization.
