# Supplementary material for: Length-of-stay and factors associated with early discharge after birth in health facilities in Guinea by mode of birth: Secondary analysis of Demographic and Health Survey 2018
Source: PLOS Glob Public Health. 2024 Oct 3;4(10):e0003786. doi: 10.1371/journal.pgph.0003786 (PMC11449310; doi:10.1371/journal.pgph.0003786)
Supplement: S1 Table — (DOCX) [file pgph.0003786.s002.docx]

**S1 Table 1.1 – Outcome variable definitions and details**

| **Indicators** | **Question code** | **Variable code in individual recode dataset** | **Variable used in analysis** |
| --- | --- | --- | --- |
| Length-of-stay (LoS) after facility birth | Q431 | m61_1 | Continuous – converted to hours based on Campbell et al 2016  Categorical – 9 categories  <2 hours; 2-3 hours; 4-5 hours; 6-12 hours; 13-24 hours; 25-72 hours; 73-96 hours; 97-168 hours; >168 hours |
| Premature discharge – WHO recommendation cut-offs | Q431 | m61_1 | Categorical – 2 categories  Coded from continuous LoS variable:  1= Premature discharge (Yes) when LoS<24 hours after vaginal birth or LoS<72 hours after caesarean section  0 = Recommended LoS (No) when LoS>=24 hours after vaginal birth or LoS>=72 hours after caesarean section |
| Premature discharge – locally informed cut-offs | Q431 | m61_1 | Categorical – 2 categories  Coded from continuous LoS variable:  1= Premature discharge (Yes) when LoS<6 hours after vaginal birth or LoS<72 hours after caesarean section  0= Recommended LoS (No) when LoS>=6 hours after vaginal birth or LoS>=72 hours after caesarean section |
| Woman received postpartum health check in health facility before discharge | Q435 | m62_1  m64_1 | Categorical – 2 categories  1= Health check received from a skilled provider (doctor, nurse/midwife, technical health officer)  0= No health checked received from a skilled provider (no check, or check provided by traditional birth attendant, community/village health worker, or other) |

**S1 Table 1.2 – Independent variables definitions and details**

| **Level** | **Variable** | **Question code** | **Variable code in IR dataset** | **Variable as used in analysis** |
| --- | --- | --- | --- | --- |
| **Community & family factors** | Region |  | v024 | Categorical – 8 categories  Boké, Conakry, Faranah, Kankan, Kindia, Labé, Mamou, Nzérékoré |
|  | Place of residence |  | v025 | Categorical – 2 categories  0= Rural  1= Urban |
|  | Ethnicity | Q123 | v131 | Categorical – 4 categories  1= Sousou  2= Peul  3= Malinké  4= Other (Kissi, Toma, Guerzé, Other, Etranger) |
|  | Marital and cohabiting status (at time of survey) | Q704 | v502 and v504 | Categorical – 2 categories  0= Not in union/not living with partner  1= In union and living with partner |
|  | Number of household members | Household  schedule | v136 (continuous) | Categorical – 3 categories  0= 2-3 members  1= 4-9 members  2= 10 or more members |
|  | Relationship to head of household | Household  schedule | v150 | Categorical – 4 categories  1= Self  2= Partner  3= Child/child in law  4= Other (grandchild, parent, brother/sister, other relative, adopted/foster child, not related, brother/sister in law) |
|  | Decision making regarding healthcare service seeking for self | Q922 | v743a | Categorical – 2 categories  0= Not involved in decision making (partner alone, someone else, other)  1= Involved in decision making partially (with husband) or completely (alone)  n = 276 missing values |
| **Facility characteristics & norms** | Type of facility of birth | Q430 | m15_1 | Categorical – 4 categories  1= Government hospital (gov hospital, regional, prefectorial)  2= Government lower level facility (gov health post, health center, other public health sector)  3= Non-governmental hospital (private hospital, clinic)  4= Non-governmental lower-level facility (clinic AGBEF, private care clinic) |
|  | Skilled attendance at birth | Q429 | m3a-n_1 | Categorical – 2 categories  0= No skilled attendance at birth (if highest skilled cadre is traditional birth attendant, or community health worker, parent/friend, neighbour, or other person)  1= Skilled attendance at birth (if highest skilled cadre is doctor or nurse/midwife or technical health officer) |
|  | Day of birth | Q215 | b17_01, b1_01, b2_01 | Categorical – 2 categories  0= Weekend (Saturday, Sunday)  1= Weekday (Monday-Friday)  Days of the week generated from day of birth using ‘*dow’* function in Stata. |
| **Women’s socio-demographic characteristics** | Maternal age at date of birth  [newborn’s date of birth – respondent’s date of birth] |  | b3_01 – v011 | Continuous - in years  [range=13.25; 48.75]  Categorical – 5 categories  0= 13-19 years  1= 20-24 years  2= 25-29 years  3= 30-34 years  4= 35-49 years |
|  | Highest completed education level | Q107-Q108 | v106 | Categorical – 3 categories  1= No education  2= Primary education  3= Secondary education or higher |
|  | Occupation frequency at time of survey | Q915 | v731, v732 | Categorical – 4 categories  0= Not employed in the past 12 months  1= Occasionally employed  2= Seasonally employed  3= Employed |
|  | Household wealth index for urban / rural |  | v190a | Categorical – 5 categories  0= Poorest  1= Poorer  2= Middle  3= Richer  4= Richest |
|  | Insurance ownership at time of survey | Q1109 | v481 | Categorical – 2 categories  0= not covered  1= covered by health insurance |
|  | Mobile phone ownership at time of survey | Q116 | v169A | Categorical – 2 categories  0= does not own  1= owns mobile phone |
|  | Perception of distance to health facility | Q1108 | v467d | Categorical – 2 categories  0= Not a big problem  1= Big problem preventing from care seeking for self |
|  | Getting permission to go see a care provider | Q1108 | v467b | Categorical – 2 categories  0= Not a big problem  1= Big problem preventing from care seeking for self |
|  | Getting money needed for treatment | Q1108 | v467c | Categorical – 2 categories  0= Not a big problem  1= Big problem preventing from care seeking for self |
|  | Not wanting to go alone | Q1108 | v4767f | Categorical – 2 categories  0= Not a big problem  1= Big problem preventing from care seeking for self |
| **Women's needs & obstetric history** | Parity at index birth | Birth history | b0_01-b0_20 | Categorical – 3 categories – number of pregnancies (multiple pregnancies counted as 1)  1= Primiparous  2= Multiparous 2-3  3= Multiparous 4 or more |
|  | ANC frequency during pregnancy | Q408  Q412 | m14_1 | Categorical – 3 categories  0= None  1= 1-3 visits  2= 4 or more visits |
|  | Timing of first ANC visit | Q408  Q411 | m13_1 | Categorical – 3 categories  0= None  1= During 1^st^ trimester  2= Beyond 1^st^ trimester |
|  | Mode of birth | Q432 | m17_1 | Categorical – 2 categories  0= Vaginal birth  1= Birth by caesarean section |
|  | Multiple births | Q214 | b0_01 | Categorical – 2 categories  0= Single birth  1= Multiple birth |
|  | Pregnancy wantedness at time of pregnancy | Q405 | m10_01 | Categorical – 2 categories  0= Wanted later or not at all  1= Wanted at time of pregnancy |
|  | Ever had a terminated pregnancy | Q206 / Q230 | v228 | Categorical – 2 categories  0= Never had a terminated pregnancy  1= Ever had a terminated pregnancy |
| **Newborn characteristics** | Newborn sex | Q213 | b4_01 | Categorical – 2 categories  0= Boy  1= Girl |
|  | Perception of newborn size at birth | Q426 | m18_1 | Categorical – 2 categories  0= Smaller than average (smaller than average, very small)  1= Average size or larger (very large, larger than average, average)  n=19 missing values |
|  | Newborn survival and time of death |  | b5_01, b6_01, m61_1 | Categorical – 3 categories  0= Survived until survey  1= Died on/before day of discharge  2= Died after discharge  Recoded following the guidance in Campbell et al 2016 |
