# Supplementary material for: Length-of-stay and factors associated with early discharge after birth in health facilities in Guinea by mode of birth: Secondary analysis of Demographic and Health Survey 2018
Source: PLOS Glob Public Health. 2024 Oct 3;4(10):e0003786. doi: 10.1371/journal.pgph.0003786 (PMC11449310; doi:10.1371/journal.pgph.0003786)
Supplement: S2 Table — (DOCX) [file pgph.0003786.s003.docx]

**S2 Table 2.1** – Characteristics of the sample including women who had outlier values on the outcome of interest (length-of-stay >3 weeks) among women who gave their most recent livebirth in a health facility in the five years preceding the Guinea DHS2018, by mode of birth (n=2,782)

|  |  | **Sensitivity including outliers** | | | | | | | | |
| --- | --- | --- | --- | --- | --- | --- | --- | --- | --- | --- |
|  |  | **Vaginal birth** | | | **Caesarean section** | | | **Total** | | |
|  | **Characteristics** | **n** | | **% [95%CI]** | **n** | **% [95%CI]** | | **n** | **% [95%CI]** | |
| **Community and family factors** | **Region** |  | | |  | | |  | | |
|  | Boké | 324 | 8.8 [7.1; 10.9] | | 25 | | 11.7 [8.9; 15.2] | 349 | | 9.01 [7.3; 11] |
|  | Conakry | 423 | 20.2 [18.1; 22.6] | | 36 | | 26.7 [22.4; 31.5] | 459 | | 20.6 [18.5; 22.9] |
|  | Faranah | 236 | 6.7 [5.5; 8] | | 15 | | 6.4 [5; 8.1] | 251 | | 6.6 [5.5; 8] |
|  | Kankan | 417 | 17.1 [14.2; 20.5] | | 13 | | 8.6 [6.4; 11.4] | 430 | | 16.6 [13.8; 19.8] |
|  | Kindia | 373 | 15 [12.7; 17.6] | | 20 | | 13.4 [11.2; 16.1] | 393 | | 14.9 [12.6; 17.4] |
|  | Labé | 219 | 6.7 [5.5; 8.2] | | 20 | | 9.3 [6.6; 12.8] | 239 | | 6.8 [5.6; 8.3] |
|  | Mamou | 191 | 5.2 [4.1; 6.5] | | 17 | | 5.7 [4.2; 7.8] | 208 | | 5.2 [4.1; 6.6] |
|  | Nzérékoré | 427 | 20.3 [17.3; 23.7] | | 26 | | 18.2 [13.1; 24.7] | 453 | | 20.2 [17.3; 23.5] |
|  | **Residence** |  |  | |  | |  |  | |  |
|  | Urban | 1244 | 46.9 [41.4; 52.5] | | 112 | | 64.7 [54.9; 73.4] | 1356 | | 48.0 [42.5; 53.5] |
|  | Rural | 1366 | 53.1 [47.5; 58.6] | | 60 | | 35.3 [26.6; 45.1] | 1426 | | 52.0 [46.5; 57.5] |
|  | **Ethnicity** |  |  | |  | |  |  | |  |
|  | Soussou | 573 | 22.2 [19.2; 25.6] | | 31 | | 19.3 [13.9; 26.2] | 604 | | 22.1 [19.1; 25.3] |
|  | Peuls | 816 | 26.6 [23.7; 29.7] | | 67 | | 34.6 [27.6; 42.5] | 883 | | 27.1 [24.2; 30.2] |
|  | Malinké | 826 | 32.2 [28.5; 36.1] | | 47 | | 28.1 [21.9; 35.2] | 873 | | 31.9 [28.4; 35.8] |
|  | Other | 395 | 19.0 [15.4; 23.1] | | 27 | | 18.0 [11.9; 26] | 422 | | 18.9 [15.4; 22.9] |
|  | **Marital and cohabiting status at time of survey** |  |  | |  | |  |  | |  |
|  | Not in union/not living with a partner | 615 | 23.1 [21.1; 25.3] | | 45 | | 26.1 [19.5; 34] | 660 | | 23.3 [21.3; 25.4] |
|  | Living with a partner | 1995 | 76.9 [74.7; 78.9] | | 127 | | 73.9 [66; 80.5] | 2122 | | 76.7 [74.6; 78.7] |
|  | **Involvement in decision making regarding own healthcare*** | 937 | 42.2 [38.7; 45.9] | | 61 | | 39.3 [31; 48.2] | 998 | | 42.1 [38.5; 45.7] |
|  | **Number of household members** |  |  | |  | |  |  | |  |
|  | 2-3 members | 213 | 8.2 [7.2; 9.4] | | 13 | | 7.0 [3.9; 12.4] | 226 | | 8.1 [7.1; 9.3] |
|  | 4-9 members | 1741 | 67.3 [64.6; 69.8] | | 115 | | 66.8 [58.3; 74.4] | 1856 | | 67.3 [64.7; 69.8] |
|  | 10 or more members | 656 | 24.5 [21.9; 27.3] | | 44 | | 26.2 [19; 34.9] | 700 | | 24.6 [22; 27.3] |
|  | **Relation to head of the household** |  |  | |  | |  |  | |  |
|  | Self | 154 | 6.1 [5.1; 7.3] | | 14 | | 7.4 [4.4; 12.2] | 168 | | 6.2 [5.2; 7.3] |
|  | Partner | 1790 | 69.0 [66.6; 71.2] | | 118 | | 70.3 [62.4; 77.1] | 1908 | | 69.0 [66.8; 71.2] |
|  | Child/child in law | 447 | 17.0 [15.3; 18.8] | | 25 | | 13.7 [9.2; 19.9] | 472 | | 16.8 [15.2; 18.6] |
|  | Other | 219 | 7.9 [6.8; 9.2] | | 15 | | 8.6 [5.1; 14.3] | 234 | | 8.0 [6.8; 9.2] |
| **Facility characteristics and norms** | **Type of facility** |  |  | |  | |  |  | |  |
|  | Government lower level facility | 1823 | 71.8 [68.5; 74.8] | | 53 | | 30.5 [22.1; 40.5] | 1876 | | 69.4 [66.1; 72.5] |
|  | Government hospital | 513 | 17.9 [15.4; 20.7] | | 101 | | 58.7 [49; 67.7] | 614 | | 20.3 [17.7; 23.1] |
|  | Non-government hospital | 203 | 7.8 [6.5; 9.4] | | 15 | | 9.5 [5.6; 15.6] | 218 | | 7.9 [6.6; 9.4] |
|  | Non-government lower level facility | 71 | 2.5 [1.8; 3.5] | | 3 | | 1.4 [0.4; 4.2] | 74 | | 2.5 [1.8; 3.4] |
|  | **Skilled attendance at birth** | 2499 | 94.8 [92.2; 96.6] | | 167 | | 95.1 [86.5; 98.3] | 2666 | | 94.8 [92.2; 96.6] |
|  | **Day of birth** |  |  | |  | |  |  | |  |
|  | Weekday | 1815 | 69.1 [67.2; 70.9] | | 124 | | 71.6 [63.8; 36.2] | 1939 | | 69.2 [67.4; 71] |
|  | Weekend | 795 | 30.9 [29.1; 32.8] | | 48 | | 28.4 [21.7; 36.2] | 843 | | 30.8 [28.9; 32.6] |
| **Women's socio-economic characteristics** | **Maternal age at birth (years; mean, se)** | 26.9 | 0.15 | | 28.1 | | 0.67 | 27 | | 0.15 |
|  | **Maternal age at birth (in years)** |  |  | |  | |  |  | |  |
|  | 13-19 years | 423 | 16.3 [14.9; 17.8] | | 23 | | 12.1 [7.8; 18.1] | 446 | | 16.0 [14.7; 17.5] |
|  | 20-24 years | 582 | 22.6 [20.8; 24.5] | | 27 | | 15.6 [10.3; 22.9] | 609 | | 22.2 [20.5; 24.1] |
|  | 25-29 years | 644 | 24.6 [23; 26.3] | | 52 | | 30.8 [24.1; 38.4] | 696 | | 25.0 [23.4; 26.6] |
|  | 30-34 years | 473 | 17.9 [16.4; 19.5] | | 34 | | 19.4 [13.3; 27.5] | 507 | | 18.0 [16.6; 19.5] |
|  | 35-49 years | 488 | 18.6 [16.9; 20.3] | | 36 | | 22.1 [15.2; 31.1] | 524 | | 18.8 [17.2; 20.4] |
|  | **Highest education level reached** |  |  | |  | |  |  | |  |
|  | No education | 1719 | 66.5 [63.9; 69.1] | | 95 | | 53.3 [45.1; 61.4] | 1814 | | 65.8 [63.2; 68.3] |
|  | Primary education | 356 | 13.0 [11.5; 14.6] | | 27 | | 15.8 [10.7; 22.6] | 383 | | 13.1 [11.7; 14.8] |
|  | Secondary or higher | 535 | 20.5 [18.4; 22.6] | | 50 | | 30.9 [24.3; 38.4] | 585 | | 21.1 [19.1; 23.3] |
|  | **Occupation frequency** |  |  | |  | |  |  | |  |
|  | Not worked in the past 12 months | 689 | 24.8 [22.5; 27.3] | | 51 | | 27.3 [20.7; 35.2] | 740 | | 25.0 [22.7; 27.4] |
|  | Occasional | 339 | 12.7 [11.1; 14.5] | | 14 | | 9.1 [5.3; 15.1] | 353 | | 12.5 [10.9; 14.3] |
|  | Seasonal | 513 | 21.0 [18.2; 24] | | 18 | | 11.6 [7.1; 18.5] | 531 | | 20.4 [17.8; 23.4] |
|  | All year | 1069 | 41.5 [38.5; 44.5] | | 89 | | 52.0 [43.7; 60.1] | 1158 | | 42.1 [39.2; 45.1] |
|  | **Household wealth index** |  |  | |  | |  |  | |  |
|  | Poorest | 455 | 16.7 [13.7; 20.2] | | 34 | | 18.1 [12.3; 25.8] | 489 | | 16.8 [13.9; 20.2] |
|  | Poorer | 517 | 19.2 [17.1; 21.4] | | 23 | | 11.8 [7.8; 17.4] | 540 | | 18.7 [16.8; 20.9] |
|  | Middle | 522 | 20.8 [18.7; 23] | | 29 | | 18.5 [12.5; 26.5] | 551 | | 20.7 [18.6; 22.8] |
|  | Richer | 538 | 21.3 [19.1; 23.8] | | 34 | | 20.9 [14.9; 28.4] | 572 | | 21.3 [19.1; 23.7] |
|  | Richest | 578 | 22.0 [18.8; 25.5] | | 52 | | 30.7 [22.5; 40.5] | 630 | | 22.5 [19.3; 25.9] |
|  | **Owns health insurance** | 46 | 1.8 [1.2; 2.7] | | 6 | | 4.0 [1.6; 9.6] | 52 | | 2.0 [1.3; 2.9] |
|  | **Owns mobile phone** | 2042 | 77.1 [74.2; 79.7] | | 158 | | 92.2 [86.6; 95.5] | 2200 | | 77.9 [75.2; 80.5] |
|  | **Issue perceived as a big problem to access healthcare** |  |  | |  | |  |  | |  |
|  | Distance to health facility | 939 | 35.4 [32.3; 38.5] | | 45 | | 23.9 [17.6; 31.6] | 984 | | 34.7 [31.8; 37.8] |
|  | Getting permission to go | 596 | 21.6 [18.9; 24.4] | | 36 | | 20.3 [14.5; 27.8] | 632 | | 21.5 [18.9; 24.3] |
|  | Getting money needed for treatment | 1456 | 55.5 [52.4; 58.5] | | 79 | | 39.2 [31.5; 47.5] | 1535 | | 54.5 [51.5; 57.5] |
|  | Not wanting to go alone | 614 | 22.9 [20.4; 25.5] | | 37 | | 19.8 [13.9; 27.3] | 651 | | 22.7 [20.3; 25.3] |
| **Women's needs and obstetrics history** | **Parity at index birth** |  |  | |  | |  |  | |  |
|  | Primiparous | 618 | 23.9 [22.2; 25.6] | | 49 | | 26.7 [20.2; 34.5] | 667 | | 24.1 [22.4; 25.8] |
|  | Multiparous 2-3 | 972 | 36.8 [34.6; 38.9] | | 67 | | 40.3 [32.9; 48.2] | 1039 | | 36.9 [34.9; 39.1] |
|  | Multiparous 4 or more | 1020 | 39.3 [37.3; 41.5] | | 56 | | 32.9 [25.4; 41.5] | 1076 | | 38.9 [36.9; 41.1] |
|  | **ANC visits during pregnancy** |  |  | |  | |  |  | |  |
|  | None | 85 | 2.9 [2.2; 3.9] | | 5 | | 3.7 [1.3; 9.9] | 90 | | 3.0 [2.2; 3.9] |
|  | 1-3 visits | 1322 | 50.5 [47.9; 53] | | 69 | | 39.4 [31.7; 47.7] | 1391 | | 49.8 [47.3; 52.4] |
|  | 4 or more visits | 1203 | 46.6 [43.9; 49.3] | | 98 | | 56.9 [48.4; 64.9] | 1301 | | 47.2 [44.6; 49.9] |
|  | **Timing of first ANC visit** |  |  | |  | |  |  | |  |
|  | None | 85 | 2.9 [2.2; 3.9] | | 5 | | 3.7 [1.3; 9.9] | 90 | | 3.0 [2.2; 3.9] |
|  | During 1^st^ trimester | 900 | 34.3 [31.5; 37.1] | | 60 | | 30.8 [23.9; 38.5] | 960 | | 34.0 [31.3; 36.9] |
|  | Beyond 1^st^ trimester | 1625 | 62.8 [59.8; 65.7] | | 107 | | 65.5 [57.6; 72.7] | 1732 | | 63.0 [60.1; 65.8] |
|  | **Multiple birth** | 75 | 2.9 [2.2; 3.6] | | 8 | | 3.8 [1.8; 7.5] | 83 | | 2.9 [2.3; 3.7] |
|  | **Pregnancy wanted at the time** | 2193 | 84.2 [82.6; 85.8] | | 147 | | 85.3 [78.3; 90.3] | 2340 | | 84.3 [82.7; 85.8] |
|  | **Ever had a terminated pregnancy** | 274 | 10.0 [8.8; 11.6] | | 36 | | 21.8 [15.6; 29.6] | 310 | | 10.8 [9.5; 12.2] |
| **Newborn characteristics** | **Newborn sex** |  |  | |  | |  |  | |  |
|  | Girl | 1263 | 48.3 [46.4; 50.2] | | 90 | | 52.4 [44.6; 60.1] | 1353 | | 48.5 [46.7; 50.4] |
|  | Boy | 1347 | 51.7 [49.8; 53.6] | | 82 | | 47.6 [39.9; 55.4] | 1429 | | 51.5 [49.6; 53.3] |
|  | **Perceived size at birth**** |  |  | |  | |  |  | |  |
|  | Smaller than average | 210 | 7.8 [6.7; 9.2] | | 19 | | 12.4 [7.6; 19.5] | 229 | | 8.1 [6.9; 9.4] |
|  | Average or larger | 2382 | 92.2 [90.8; 93.3] | | 152 | | 87.6 [80.5; 92.4] | 2534 | | 91.9 [90.6; 93] |
|  | **Newborn survival** |  |  | |  | |  |  | |  |
|  | Survived until survey | 2485 | 95.1 [94; 96.1] | | 161 | | 93.9 [88.2; 96.9] | 2646 | | 95 [93.9; 96] |
|  | Died on /before discharge | 21 | 0.7 [0.4; 1.1] | | 5 | | 3.8 [1.4; 9.8] | 26 | | 0.9 [0.6; 1] |
|  | Died after discharge | 104 | 4.2 [3.3; 5.3] | | 6 | | 2.3 [0.9; 5.6] | 110 | | 4.1 [3.2; 5.1] |
| **Total** | | 2610 | 94.2 [93.1; 95.1] | | 172 | | 5.8 [4.9; 6.9] | 2782 | | 100 |

**S2 Table 2.2** – Postpartum length-of-stay (both continuous and categorical) among women who gave their most recent livebirth in a health facility in the five years preceding the Guinea DHS2018 including women who had outlier values (>3 weeks) for postpartum length-of-stay, by mode of birth (n=2,782)

|  | **With outliers** | | | | | |
| --- | --- | --- | --- | --- | --- | --- |
|  | **Vaginal birth** | | **Birth by Caesarean section** | | **Total** | |
|  | **n** | **% [95% CI]** | **n** | **% [95% CI]** | **n** | **% [95% CI]** |
| **Length-of-stay**  **[mean (se), median (iqr)]** | 15.6 (4.2) | 3 (2-4) | 182 (20.1) | 132 (60-252) | 25.2 (4.1) | 3 (2-5) |
| **Length-of-stay (categorical)** |  |  |  |  |  |  |
| **<6 hours** | **2072** | **81.3 [79.4; 83.0]** | **29** | **18.9 [11.5; 29.6]** | **2101** | **77.7 [75.7; 79.5]** |
| <2 hours | 534 | 20.3 [18.3; 22.5] | 5 | 3.3 [1.3; 8.1] | 539 | 19.3 [17.4; 21.5] |
| 2-3 hours | 1165 | 46.9 [44.3; 49.5] | 18 | 12.6 [6.3; 23.4] | 1183 | 44.9 [42.3; 47.5] |
| 4-5 hours | 373 | 14.1 [12.6; 15.7] | 6 | 3.1 [1.1; 8.1] | 379 | 13.4 [12.0; 14.9] |
| **6-23 hours** | **249** | **9.1 [7.8; 10.5]** | **2** | **0.8 [0.2; 3.8]** | **251** | **8.6 [7.4; 9.9]** |
| 6-12 hours | 216 | 7.3 [6.2; 8.5] | 2 | 0.8 [0.2; 3.8] | 218 | 6.9 [5.9; 8.1] |
| 13-23 hours | 33 | 1.2 [0.8; 2.7] | 0 | 0 | 33 | 1.2 [0.8; 1.6] |
| **≥24 hours** | **289** | **10.2 [8.9; 11.6]** | **141** | **80.2 [69.7; 87.8]** | **430** | **14.3 [12.9; 15.8]** |
| 24-71 hours | 237 | 8.4 [7.0; 9.7] | 10 | 6.3 [3.2; 12.1] | 247 | 8.3 [7.2; 9.6] |
| 72-95 hours | 18 | 0.6 [0.3; 1.1] | 27 | 14.3 [9.3; 21.2] | 45 | 1.4 [0.9; 1.9] |
| 96-167 hours | 13 | 0.5 [0.3; 0.9] | 39 | 22.9 [16.5; 30.9] | 52 | 1.8 [1.3; 2.4] |
| ≥168 hours | 21 | 0.7 [0.4; 1.2] | 65 | 36.8 [29.0; 45.3] | 86 | 2.8 [2.2; 3.5] |
| **WHO recommended cut-offs^†^** |  |  |  |  |  |  |
| Recommended LoS | 289 | 10.2 [8.9; 11.6] | 131 | 73.9 [63.6; 82.1] | 420 | 13.9 [12.6; 15.4] |
| Premature discharge | 2321 | 89.8 [88.4; 91] | 41 | 26.1 [17.9; 36.4] | 2362 | 86.1 [84.6; 87.4] |
| **Locally informed cut-offs^‡^** |  |  |  |  |  |  |
| Recommended LoS | 538 | 18.72 [16.9; 20.6] | 131 | 73.9 [63.6; 82.1] | 669 | 21.9 [20.1; 23.8] |
| Premature discharge | 2072 | 81.28 [79.4; 83.0] | 41 | 26.1 [17.9; 36.4] | 2113 | 78.1 [76.2; 79.9] |
| ^†^Premature discharge before 24hrs for vaginal births and before 72hrs for c-section | | | | | | |
| ^‡^Premature discharge before 6hrs for vaginal births and before 72hrs for c-section | | | | | | |
