# Supplementary material for: Length-of-stay and factors associated with early discharge after birth in health facilities in Guinea by mode of birth: Secondary analysis of Demographic and Health Survey 2018
Source: PLOS Glob Public Health. 2024 Oct 3;4(10):e0003786. doi: 10.1371/journal.pgph.0003786 (PMC11449310; doi:10.1371/journal.pgph.0003786)
Supplement: S3 Table — (DOCX) [file pgph.0003786.s004.docx]

**S3 Table 3.1** – Comparison of the characteristics of the included sample and the sample of women with missing values on postpartum length-of-stay among women who gave their most recent livebirth in a health facility in the five years preceding the Guinea DHS2018 (n=2,763 vs n=99)

|  |  | **Included sample** | | **Missing values on LoS** | | **p-value** |
| --- | --- | --- | --- | --- | --- | --- |
|  | **Characteristics** | **n** | **% [95%CI]** | **n** | **% [95%CI]** |  |
| **Community and family factors** | **Region** |  |  |  |  | <0.001 |
|  | Boké | 343 | 8.9 [7.2; 11] | 10 | 5.9 [4.1; 8.6] |  |
|  | Conakry | 453 | 20.4 [18.3; 22.7] | 8 | 9.3 [6.8; 12.6] |  |
|  | Faranah | 250 | 6.7 [5.5; 8] | 6 | 5.7 [1.9; 15.8] |  |
|  | Kankan | 430 | 16.7 [13.9; 20] | 12 | 9.9 [7.1; 13.8] |  |
|  | Kindia | 390 | 14.9 [12.6; 17.4] | 6 | 5.6 [3.2; 9.6] |  |
|  | Labé | 237 | 6.9 [5.6; 8.3] | 5 | 3.2 [2.1; 4.7] |  |
|  | Mamou | 207 | 5.2 [4.1; 6.6] | 27 | 19.2 [13.8; 26.2] |  |
|  | Nzérékoré | 453 | 20.3 [17.4; 23.6] | 25 | 41.1 [29.3; 54.1] |  |
|  | **Residence** |  |  |  |  | <0.001 |
|  | Urban | 1343 | 47.8 [42.3; 53.3] | 29 | 23.6 [15.3; 34.5] |  |
|  | Rural | 1420 | 52.2 [46.6; 57.7] | 70 | 76.4 [65.6; 84.7] |  |
|  | **Ethnicity** |  |  |  |  | <0.001 |
|  | Soussou | 600 | 22.1 [19.3; 25.3] | 11 | 8.1 [4.3; 14.6] |  |
|  | Peuls | 871 | 26.8 [23.9; 29.9] | 42 | 31.9 [23.2; 41.9] |  |
|  | Malinké | 871 | 32.1 [28.5; 35.9] | 18 | 16.1 [10.4; 24.2] |  |
|  | Other | 421 | 19.0 [15.5; 23.1] | 28 | 43.9 [32.4; 56.1] |  |
|  | **Marital and cohabiting status at time of survey** |  |  |  |  | 0.122 |
|  | Not in union/not living with a partner | 655 | 23.3 [21.3; 25.4] | 30 | 31.2 [21; 43.5] |  |
|  | Living with a partner | 2108 | 76.7 [74.6; 78.7] | 69 | 68.8 [56.5; 78.9] |  |
|  | **Involvement in decision making regarding own healthcare*** | 995 | 42.3 [38.7; 46] | 43 | 57.4 [43.7; 70] | 0.037 |
|  | **Number of household members** |  |  |  |  | 0.046 |
|  | 2-3 members | 223 | 8.1 [7.1; 9.3] | 2 | 1.4 [0.3; 6] |  |
|  | 4-9 members | 1842 | 67.2 [64.6; 69.7] | 69 | 73.9 [62.9; 82.6] |  |
|  | 10 or more members | 698 | 24.7 [22.1; 27.4] | 28 | 24.7 [16.2; 35.6] |  |
|  | **Relation to head of the household** |  |  |  |  | 0.092 |
|  | Self | 167 | 6.2 [5.3; 7.3] | 3 | 2.3 [0.7; 7.5] |  |
|  | Partner | 1893 | 68.9 [66.7; 71.1] | 63 | 63.5 [51.6; 73.9] |  |
|  | Child/child in law | 469 | 16.8 [15.2; 18.6] | 24 | 25.6 [17.4; 35.9] |  |
|  | Other | 234 | 8.1 [6.9; 9.3] | 9 | 8.6 [3.9; 17.9] |  |
| **Facility characteristics and norms** | **Type of facility** |  |  |  |  | 0.039 |
|  | Government lower level facility | 1870 | 69.6 [66.3; 72.7] | 79 | 83.6 [73; 90.6] |  |
|  | Government hospital | 605 | 20.1 [17.6; 22.9] | 14 | 11.6 [5.6; 22.7] |  |
|  | Non-government hospital | 214 | 7.8 [6.5; 9.3] | 3 | 2.1 [0.6; 7.0] |  |
|  | Non-government lower level facility | 74 | 2.5 [1.8; 3.4] | 3 | 2.6 [0.8; 8.1] |  |
|  | **Skilled attendance at birth** | 2647 | 94.8 [92.2; 96.6] | 96 | 94.6 [84.1; 98.3] | 0.928 |
|  | **Day of birth** |  |  |  |  | 0.612 |
|  | Weekday | 1925 | 69.2 [67.3; 71] | 68 | 66.8 [56.7; 75.6] |  |
|  | Weekend | 838 | 30.8 [29; 32.7] | 31 | 33.1 [24.4; 43.3] |  |
| **Women's socio-economic characteristics** | **Maternal age at birth (in years; mean, se)** | 27 | 0.15 [26.7; 27.3] | 27.8 | 0.64 [26.6; 29.1] |  |
|  | **Maternal age at birth (in years)** |  |  |  |  | 0.06 |
|  | 13-19 years | 444 | 16.1 [14.7; 17.5] | 10 | 9.4 [4.7; 18] |  |
|  | 20-24 years | 605 | 22.2 [20.5; 24] | 20 | 19.5 [12.1; 29.7] |  |
|  | 25-29 years | 690 | 24.9 [23.4; 26.6] | 32 | 37.2 [27.3; 48.3] |  |
|  | 30-34 years | 503 | 18.0 [16.5; 19.5] | 14 | 13.3 [7.5; 22.4] |  |
|  | 35-49 years | 521 | 18.8 [17.2; 20.4] | 23 | 20.6 [13.5; 30.2] |  |
|  | **Highest education level reached** |  |  |  |  | 0.041 |
|  | No education | 1802 | 65.8 [63.2; 68.3] | 79 | 80.9 [69.6; 88.8] |  |
|  | Primary education | 380 | 13.1 [11.6; 14.7] | 9 | 8.1 [3.6; 17.5] |  |
|  | Secondary or higher | 581 | 21.1 [19.1; 23.3] | 11 | 10.9 [5.6; 20.2] |  |
|  | **Occupation frequency** |  |  |  |  | 0.009 |
|  | Not worked in the past 12 months | 730 | 24.8 [22.5; 27.3] | 35 | 25.1 [17.1; 35.4] |  |
|  | Occasional | 349 | 12.4 [10.8; 14.2] | 8 | 6.8 [3.2; 13.9] |  |
|  | Seasonal | 530 | 20.6 [17.9; 23.5] | 29 | 42.3 [27.5; 58.5] |  |
|  | All year | 1154 | 42.2 [39.3; 45.2] | 27 | 25.8 [15.6; 39.4] |  |
|  | **Household wealth index** |  |  |  |  | 0.833 |
|  | Poorest | 481 | 16.7 [13.8; 20.1] | 15 | 13.1 [6.1; 25.8] |  |
|  | Poorer | 539 | 18.8 [16.8; 20.9] | 20 | 21.7 [13.9; 32.3] |  |
|  | Middle | 550 | 20.8 [18.7; 22.9] | 24 | 23.6 [16.3; 32.9] |  |
|  | Richer | 567 | 21.2 [19; 23.6] | 18 | 22.7 [15.8; 31.4] |  |
|  | Richest | 626 | 22.5 [19.3; 25.9] | 22 | 18.9 [11.8; 29.1] |  |
|  | **Owns health insurance** | 52 | 2.0 [1.3; 2.97] | 1 | 0.7 [0.09; 4.7] | 0.265 |
|  | **Owns mobile phone** | 2182 | 77.9 [75.1; 80.4] | 70 | 66.1 [54.9; 75.8] | 0.021 |
|  | **Issue perceived as a big problem to access healthcare** |  |  |  |  |  |
|  | Distance to health facility | 976 | 34.6 [62.3; 68.3] | 39 | 47.0 [33.1; 61.3] | 0.088 |
|  | Getting permission to go | 625 | 21.4 [18.8; 24.2] | 15 | 13.8 [7.9; 22.9] | 0.098 |
|  | Getting money needed for treatment | 1522 | 54.4 [51.4; 57.4] | 52 | 58.6 [45.5; 70.5] | 0.539 |
|  | Not wanting to go alone | 645 | 22.6 [20.2; 25.2] | 23 | 20.5 [12.9; 31] | 0.653 |
| **Women's needs and obstetrics history** | **Parity at index birth** |  |  |  |  | 0.003 |
|  | Primiparous | 663 | 24.1 [22.4; 25.8] | 13 | 10.5 [6.2; 17.4] |  |
|  | Multiparous 2-3 | 1029 | 36.8 [34.8; 38.9] | 42 | 47.0 [32.9; 61.5] |  |
|  | Multiparous 4 or more | 1071 | 39.1 [37.1; 41.2] | 44 | 42.5 [30.4; 55.5] |  |
|  | **ANC visits during pregnancy** |  |  |  |  | 0.023 |
|  | None | 88 | 2.9 [2.2; 3.8] | 5 | 4.1 [1.6; 20.4] |  |
|  | 1-3 visits | 1382 | 49.8 [47.3; 52.3] | 60 | 65.7 [53.6; 76.1] |  |
|  | 4 or more visits | 1293 | 47.3 [44.6; 49.9] | 34 | 30.2 [20.4; 42.2] |  |
|  | **Timing of first ANC visit** |  |  |  |  | 0.265 |
|  | None | 88 | 2.9 [2.2; 3.8] | 5 | 4.1 [1.2; 10.4] |  |
|  | During 1^st^ trimester | 953 | 34.0 [31.3; 36.8] | 34 | 26.5 [19.1; 35.5] |  |
|  | Beyond 1^st^ trimester | 1722 | 63.1 [60.1; 65.9] | 60 | 69.4 [59.4; 77.9] |  |
|  | **Mode of birth**** |  |  |  |  | 0.1682 |
|  | Vaginal birth | 2603 | 94.5 [93.5; 95.4] | 69 | 97.8 [91.2; 99.5] |  |
|  | Birth by caesarean section | 160 | 5.5 [4.5; 6.5] | 2 | 2.2 [0.5; 8.8] |  |
|  | **Multiple birth** | 80 | 2.8 [2.2; 3.6] | 3 | 3.0 [0.9; 9.7] | 0.951 |
|  | **Pregnancy wanted at the time** | 2324 | 84.3 [82.6; 85.8] | 83 | 82.7 [72.4; 89.7] | 0.712 |
|  | **Ever had a terminated pregnancy** | 307 | 10.7 [9.4; 12.2] | 6 | 8.3 [3.2; 19.9] | 0.564 |
| **Newborn characteristics** | **Newborn sex** |  |  |  |  | 0.555 |
|  | Girl | 1343 | 48.5 [46.6; 50.4] | 45 | 44.4 [31.1; 58.6] |  |
|  | Boy | 1420 | 51.5 [49.2; 53.4] | 54 | 55.6 [41.4; 68.9] |  |
|  | **Perceived size at birth***** |  |  |  |  | 0.992 |
|  | Smaller than average | 225 | 8.1 [6.9; 9.4] | 8 | 8.1 [3.5; 17.7] |  |
|  | Average or larger | 2519 | 91.9 [90.6; 93.1] | 91 | 91.8 [82.3; 96.5] |  |
|  | **Newborn survival** |  |  |  |  |  |
|  | Survived until survey | 2630 | 95.0 [93.9; 96] | 92 | 92.6 [85.3; 96.5] | 0.042 |
|  | Died on /before discharge | 25 | 0.9 [0.6; 1.3] | 4 | 3.8 [1.3; 10.8] |  |
|  | Died after discharge | 108 | 4.1 [3.2; 5.1] | 3 | 3.6 [1.2; 10.5] |  |
| **Total** | | **2763** | **100** | **99** | **100** |  |
|  | *276 missing values among included sample; 7 missing values among sample with missing LoS | | | | | |
|  | **28 missing values among sample with missing LoS | | |  |  |  |
|  | ***19 missing values among included sample |  |  |  |  |  |

**S3 Table 3.2** – Comparison of the multi-variable logistic regression models with (n=2,603) and without (n=2,585) “Perceived size at birth” as a predictor variable

|  | **Early discharge <6 hours after vaginal birth** | | | |
| --- | --- | --- | --- | --- |
|  | **Without “perceived size at birth” n=2,585** | | **With “perceived size at birth” n=2,603** | |
| **Characteristics** | **aOR [95%CI]** | **p-value** | **aOR [95%CI]** | **p-value** |
| **Region** |  |  |  |  |
| Boké | ref |  | ref |  |
| Conakry | 3.60 [2.16; 6.01] | <0.001* | 3.5 [2.08; 5.77] | <0.001* |
| Faranah | 1.98 [1.07; 3.68] | 0.031* | 2.0 [1.07; 3.71] | 0.029* |
| Kankan | 4.04 [2.10; 7.78] | <0.001* | 4.0 [2.08; 7.66] | <0.001* |
| Kindia | 2.83 [1.72; 4.65] | <0.001* | 2.8 [1.74; 4.67] | <0.001* |
| Labé | 1.01 [0.57; 1.79] | 0.984 | 1.0 [0.59; 1.83] | 0.905 |
| Mamou | 1.71 [0.93; 3.15] | 0.081 | 1.8 [1.00; 3.30] | 0.051 |
| Nzérékoré | 4.72 [2.39; 9.34] | <0.001* | 4.4 [2.24; 8.63] | <0.001* |
| **Ethnicity** |  |  |  |  |
| Soussou | ref |  | ref |  |
| Peuls | 0.96 [0.66; 1.39] | 0.825 | 0.9 [0.63; 1.33] | 0.637 |
| Malinké | 0.91 [0.59; 1.38] | 0.650 | 0.9 [0.60; 1.38] | 0.661 |
| Other (Kissi, Toma, Guerzé) | 0.83 [0.45; 1.52] | 0.456 | 0.8 [0.45; 1.48] | 0.502 |
| **Marital and cohabiting status** |  |  |  |  |
| Not in union/not living with a partner | ref |  | ref |  |
| Living with a partner | 1.08 [0.81; 1.43] | 0.611 | 1.1 [0.82; 1.43] | 0.574 |
| **Type of facility** |  |  |  |  |
| Government lower level facility | ref |  | ref |  |
| Government hospital | 0.76 [0.56; 1.04] | 0.085 | 0.8 [0.57; 1.04] | 0.090 |
| Non-government lower level facility | 2.12 [0.95; 4.76] | 0.066 | 1.9 [0.86; 4.07] | 0.114 |
| Non-government hospital | 0.55 [0.35; 0.85] | 0.008* | 0.6 [0.36; 0.87] | 0.009* |
| **Highest education level reached** |  |  |  |  |
| No education | ref |  | ref |  |
| Primary education | 0.74 [0.53; 1.02] | 0.065 | 0.8 [0.55; 1.05] | 0.095 |
| Secondary or higher | 0.76 [0.57; 1.02] | 0.065 | 0.8 [0.59; 1.05] | 0.101 |
| **Occupation frequency at time of survey** |  |  |  |  |
| Not worked in the past 12 months | ref |  | ref |  |
| Occasional | 0.97 [0.67; 1.39] | 0.854 | 1.0 [0.66; 1.38] | 0.817 |
| Seasonal | 1.09 [0.77; 1.55] | 0.604 | 1.1 [0.76; 1.50] | 0.703 |
| All year | 1.07 [0.80; 1.43] | 0.651 | 1.1 [0.81; 1.44] | 0.620 |
| **Getting permission to go** |  |  |  |  |
| Not a problem | ref |  | ref |  |
| Big problem | 0.89 [0.66; 1.21] | 0.473 | 0.8 [0.63; 1.14] | 0.275 |
| **Not wanting to go alone** |  |  |  |  |
| Not a problem | ref |  | ref |  |
| Big problem | 0.95 [0.71; 1.28] | 0.737 | 0.9 [0.67; 1.19] | 0.446 |
| **Parity at index birth** |  |  |  |  |
| Primiparous | ref |  | ref |  |
| Multiparous 2-3 | 1.18 [0.87; 1.61] | 0.269 | 1.2 [0.9; 1.64] | 0.209 |
| Multiparous 4 or more | 1.04 [0.77; 1.4] | 0.782 | 1.0 [0.78; 1.38] | 0.807 |
| **Timing of first ANC visit** |  |  |  |  |
| None | ref |  | ref |  |
| During 1^st^ trimester | 0.65 [0.35; 1.19] | 0.163 | 0.6 [0.33; 1.12] | 0.108 |
| Beyond 1^st^ trimester | 0.87 [0.47; 1.62] | 0.669 | 0.8 [0.45; 1.57] | 0.592 |
| **Birth multiplicity** |  |  |  |  |
| Singleton birth | ref |  | ref |  |
| Multiple birth | 0.54 [0.31; 0.94] | 0.030* | 0.5 [0.30; 0.91] | 0.021* |
| **Pregnancy wanted at the time** |  |  |  |  |
| Not wanted or wanted later | ref |  | ref |  |
| Wanted at the time of pregnancy | 1.25 [0.95; 1.65] | 0.117 | 1.2 [0.94; 1.63] | 0.133 |
| **Ever had a terminated pregnancy** |  |  |  |  |
| No | ref |  | ref |  |
| Yes | 0.79 [0.57; 1.09] | 0.150 | 0.8 [0.59; 1.11] | 0.180 |
| **Newborn sex** |  |  |  |  |
| Girl | 1.2 [0.57; 1.09] | 0.150 | 1.2 [0.96; 1.48] | 0.117 |
| Boy | ref |  | ref |  |
| **Perceived size at birth** |  |  |  |  |
| Smaller than average | ref |  | N/A | N/A |
| Average or larger | 1.26 [0.89; 1.8] | 0.195 |  |  |
