# Supplementary material for: Length-of-stay and factors associated with early discharge after birth in health facilities in Guinea by mode of birth: Secondary analysis of Demographic and Health Survey 2018
Source: PLOS Glob Public Health. 2024 Oct 3;4(10):e0003786. doi: 10.1371/journal.pgph.0003786 (PMC11449310; doi:10.1371/journal.pgph.0003786)
Supplement: Attachment — Submitted filename: Response to comments from reviewer 1_LB.docx [file pgph.0003786.s005.docx]

We thank the reviewer for their feedback and we appreciate the comments that we received. We believe that the suggested changes improved the structure and the flow of the manuscript. We have integrated the changes into the main-text of the paper and we provide a point-by-point response below.

1. **The introduction could benefit from a clearer structure. Consider breaking it into smaller, titled subsections (e.g., "Global Context", "Local Context", "Study Objectives") to improve readability.**

Thanks for this suggestion. We agree about the “*Objectives*” subtitles and we have made the changes in the main-text accordingly on page 4 line 92. However we believe that adding the subtitles related to the “Global context” and “Local context” would interrupt the flow of this short introduction section. After we integrated the comments that the reviewer suggested below (comments 2, 3 and 5), we believe the introduction section has become more clearly structured, and consider these changes to be sufficient.

2. **While the introduction mentions the lack of sufficient evidence informing postnatal care models, it could more explicitly highlight specific gaps that this study aims to fill. This would strengthen the rationale for the study.**

Thanks for this comment. The study aims to contribute to the knowledge available on the length-of-stay element of the PNC model in Guinea. This was specified in the first sentence of the objectives’ statement (page 4 line 93-94).

3. **Some paragraphs transition abruptly. For example, the shift from discussing global guidelines to specific barriers in Guinea could be smoother. Adding transitional sentences can help maintain a logical flow.**

Thank you, we agree and we have updated the text accordingly on pages 3-4.

4. **Please ensure that all claims are backed by appropriate citations. For instance, when discussing barriers to postnatal care in Guinea, ensure each point is referenced.**

We agree and we have added citations to support the statements on barriers to PNC in Guinea.

5. **Some sentences are long and complex. Breaking them into shorter, clearer sentences would enhance readability.**

Thank you for this remark. We have shortened/split several sentences in the introduction section as recommended and we believe this improves the clarity of the text.

6. **While the handling of missing data is explained, it would be beneficial to provide a more detailed justification for excluding certain data and how it might impact the study’s findings. Sensitivity analyses are mentioned but could be elaborated upon.**

The exclusion of the observations with missing data on length-of-stay was a limitation resulting from the data available in the secondary data and the decision was driven by the lack of available information on the outcome of the study (length-of-stay). The results of the sensitivity analysis show that differential missingness exists to a certain extent in terms of region (majority from Mamou and Nzérékoré, predominantly rural regions), residence (majority in rural settings), type of facility where birth took place, education, occupation, mobile phone ownership, and higher parity (page 8 line 195-198). We acknowledge this limitation as a potential data bias in the discussion on page 23-24 line 148-152.

7. **The explanation of statistical methods (e.g., logistic regression) is clear, but could be enhanced with more details on how potential confounders are controlled for and how model assumptions are checked.**

The factors included in the logistic regression were chosen based on the conceptual framework from Campbell et al, and on data availability in the DHS data (page 7 line 159-173). The factors with a p-value<0.2 in the simple logistic regression with the outcome were chosen to be accounted for as potential “confounders” in the multivariable logistic regression model (page 7 line 182-185). We checked for the models’ assumptions including model specification and variance inflation factor, which proved that the assumptions were met. This was added to the Methods and to the Results as footnotes to Tables 3 and 4.

8. **Please provide more information on how participant confidentiality and data protection were ensured, especially given the secondary nature of the data analysis.**

Thank you, we specified on page 8 line 210-211 that the obtained secondary data were anonymised which protects the confidentiality of participants.

9. **Please ensure all claims are well-supported with references.**

We have updated the main-text of the manuscript by providing additional references as recommended.

10. **Results: Use conventional methods of reporting results, e.g. Mean (SD), IQR, etc.**

Thanks for this recommendation, we edited the results’ section accordingly on page 9 line 226-234.
