# Supplementary material for: Length-of-stay and factors associated with early discharge after birth in health facilities in Guinea by mode of birth: Secondary analysis of Demographic and Health Survey 2018
Source: PLOS Glob Public Health. 2024 Oct 3;4(10):e0003786. doi: 10.1371/journal.pgph.0003786 (PMC11449310; doi:10.1371/journal.pgph.0003786)
Supplement: Attachment — Submitted filename: Response to reviewer 2 and editor.docx [file pgph.0003786.s007.docx]

We thank the reviewer and the editor for the feedback. We have made the required changes including adding the sub-headings to the abstract and updating the STROBE checklist (attached). We welcome the reviewer’s positive feedback to our previous response. Accordingly, we made sure to remove the term ‘prevalence’ from the entire manuscript in agreement with the reviewer.

Sincerely,

Aline Semaan on behalf of the authors
